# Supplementary material for: Willingness to pay for antiretroviral therapy, viral load, and premium services; A contingent valuation survey of people living with HIV in southern Nigeria
Source: PLoS One. 2023 Nov 16;18(11):e0289507. doi: 10.1371/journal.pone.0289507 (PMC10653503; doi:10.1371/journal.pone.0289507)
Supplement: Attachment — Submitted filename: Reviewer comments_Plosone 06-05-2023.docx [file pone.0289507.s002.docx]

**Lines 80 – 2:** The goal is not to only attain UNAIDS targets but to attain HIV epidemic control. Consider adding this caveat.

**Lines 146 – 7:** pharmacy, lab services and longer consultation times cannot be considered as premium services. These should be provided to all clients if there is need. This may raise serious ethical concerns. Please revise the sentence and clearly define what you meant by premium service.

**Line 162:** the range 18,500NGN – 100,000NGN is very wide and may mask some details. Would you to base this categorization on minimum wage, average wage and above average based on Nigeria context instead of just for convenience?

**Lines 196 -7:** Figures should be represented at the end while tables should come after the 1^st^ paragraph where they are mentioned.

**Lines 203 – 10:** You presented WTP for discrete services only. Will be good to know how many clients will be willing to pay for all the services. Additionally, information about clients willing to pay for consultation, ARV and VL or VL and ARV only e.t.c. may be helpful in prioritizing which service combination will be most affordable. Additionally, the authors didn’t establish the standard cost of the services, or are the initiated prices in Fig 1 the standard costs of these services? For example, what will be the market value of a month-dose of ARVs in the study states? Even though in line 280 4.85USD has been quoted as monthly cost of TLD, is this market price in the study states or subsidized price being supplied to the SFI sites?

**Line 295:** It will be good to know how many total clients are receiving care in the 92 selected sites and what proportion is the 1781 clients. How the sample size of 1781 clients were determined. This is important for the inference made in line 295 -6. Perhaps you can elaborate on the appropriate section in your methods.

**Line 324:** better to state that the contents are the responsibility of the authors and not FHI360.
